# Absolute and Relative Quantification of Glycation on Biotherapeutic IgGs Using Heavy Isotope–Labeled Proteins

**DOI:** 10.7171/001c.165918

**Published:** 2026-09-01

**Authors:** Sonal Priya, Marla Popov, Ron Orlando

**Affiliations:** 1 Complex Carbohydrate Research Center University of Georgia; 2 Department of Chemistry University of Georgia; 3 GlycoScientific (United States); 4 Department of Biochemistry & Molecular Biology University of Georgia

**Keywords:** Glycation, Quantitation, Heavy isotope-labeled internal standard, Liquid Chromotography-Mass Spectrometry, LC-MS, Hydrophilic Interaction Liquid Chromatography, HILIC, Immunoglobulins, IgG, Biotherapeutics

## Abstract

Post-translational modifications (PTMs), such as glycation, can be introduced during the manufacturing and storage of therapeutic proteins, including monoclonal antibodies (mAbs). Glycation modification may alter the safety and efficacy of therapeutic drugs, thus necessitating their accurate quantification. Relative quantitation is typically performed by comparing the peak area of the glycated peptide with that of its unmodified counterparts. The accuracy of this method depends on the unmodified and modified peptides having similar ionization efficiencies, as well as on the selection of the native peptide used for quantitation. We propose using non-glycated, heavy-isotope-labeled proteins to quantify glycation. The heavy isotope-labeled proteins can provide both the relative and absolute level of glycation by comparing the peak area of the unmodified tryptic peptides to that of the heavy isotope-labeled variants. To develop this approach, glycation was intentionally induced in an immunoglobulin (IgG) to have detectable glycation. A heavy isotope-labeled variant of the IgG (containing ^13^C and ^15^N lysine and arginine residues) was added to serve as an internal standard. Further, this mixture was subjected to trypsin digestion, and a bottom-up approach using Hydrophilic Interaction Liquid Chromatography (HILIC)-MS was performed. The light/heavy ratio of tryptic peptides with and without a glycation site in the forced glycation sample was monitored. Any decrease in the light/heavy ratio of peptides containing a glycation site relative to the expected ratio was attributed to the production of glycated variants. Glycation quantitation in IgGs such as Adalimumab (IgG1) and Natalizumab (IgG4) was performed. We expect this approach will apply to other PTMs.

## Introduction

Glycation is an important post-translational modification (PTM) that has been linked to diabetes, Alzheimer’s, cataract, and rheumatoid arthritis.^1,2^ It involves a reaction between a reducing sugar (glucose, galactose, fructose) and the α-amine terminal of a protein or ε-amine group on a lysine side chain.^3–6^ An unstable Schiff’s base intermediate is produced in this reaction that further rearranges into a more stable ketoamine product (Amadori product). Over time, glycated proteins in the presence of reactive intermediaries can degrade into advanced glycation end products (AGEs) that have been associated with various pathological conditions, including Alzheimer’s disease.^7–9^ Glycation is considered one of the critical quality attributes (CQAs) for biotherapeutics, as it can impact the efficacy, stability, and half-life of biotherapeutic drug products.^10–13^ There is a growing need to develop sensitive methods for detecting glycation, as regulatory filings demand detailed characterizations of the structural and chemical heterogeneity of recombinant antibodies.^14^

Several approaches to detect and quantify glycation have been reported in the literature. These methods include quantifying common AGEs such as carboxymethyl-lysine (CML), carboxyethyl-lysine (CEL), pyralline (Pyr), pentosidine (Pento-s), and arg-pyrimidine (Arg-p) using enzyme-linked immunosorbent assay (ELISA),^15–17^ high-performance liquid chromatography–fluorescence detection (HPLC–FLD), liquid chromatography–tandem mass spectrometry (LC-MS/MS), and gas chromatography–mass spectrometry (GC-MS).^13,18,19^ Further, LC-MS detection of more stable Amadori products is performed to determine information about the glycation site on a protein.^20^ However, characterizing glycation can be challenging because it may be distributed across the entire protein, often resulting in low levels of glycation at individual sites.^21^ Furthermore, glycation is known to hinder trypsin’s activity at the C-terminus of a lysine residue.^20,22^ A glycated peptide that is bound to a sugar molecule does not contain a positive charge at the primary amine to fit into the negatively charged trypsin pocket, causing a loss of enzymatic activity. Thus, a missed tryptic cleavage at a lysine residue, with a +162 Da shift in MS, is considered an indicator of a glycated peptide. Relative quantitation for glycation PTM is typically performed by dividing the peak area of the glycated missed-cleavage peptide by the sum of the peak areas of the glycated peptide and the tryptic unmodified peptide constituting the glycated missed-cleavage peptide.^21,23,24^ An alternative method for quantifying glycation at a particular site is to compare the peak area of a tryptic peptide containing the glycation site with that of a peptide within the same protein that is insensitive to glycation.^25^ Quantitation has also been attempted by first performing in vitro glycation of a protein with [^13^C_6_]-glucose and then determining the ratio between the peak areas corresponding to the peptides labeled with [^12^C_6_]-glucose and those labeled with [^13^C_6_]-glucose.^25^ The peptides glycated with [^12^C_6_]-glucose in this study were indicative of their concentration in the biological sample. Other quantitation strategies include introducing stable isotopes into glycated peptides via sodium borodeuteride reduction^26^ and spiking samples with isotope-labeled peptides.^27^

Quantitation approaches that rely on peak area of glycated peptides^23^ assume that the ionization efficiency of longer glycated missed-cleavage peptides, where the positive charge at the primary amine is neutralized due to glycation, is comparable to that of tryptic peptides. The selection of the tryptic peptide that constitutes the glycated missed-cleavage peptide is also critical, as it can affect the method’s accuracy. Additionally, these approaches require detectable levels of glycated peptides for precise quantitation and often require an enrichment step, such as boronate affinity chromatography,^10,28^ before LC-MS analysis.

**Figure 1.**
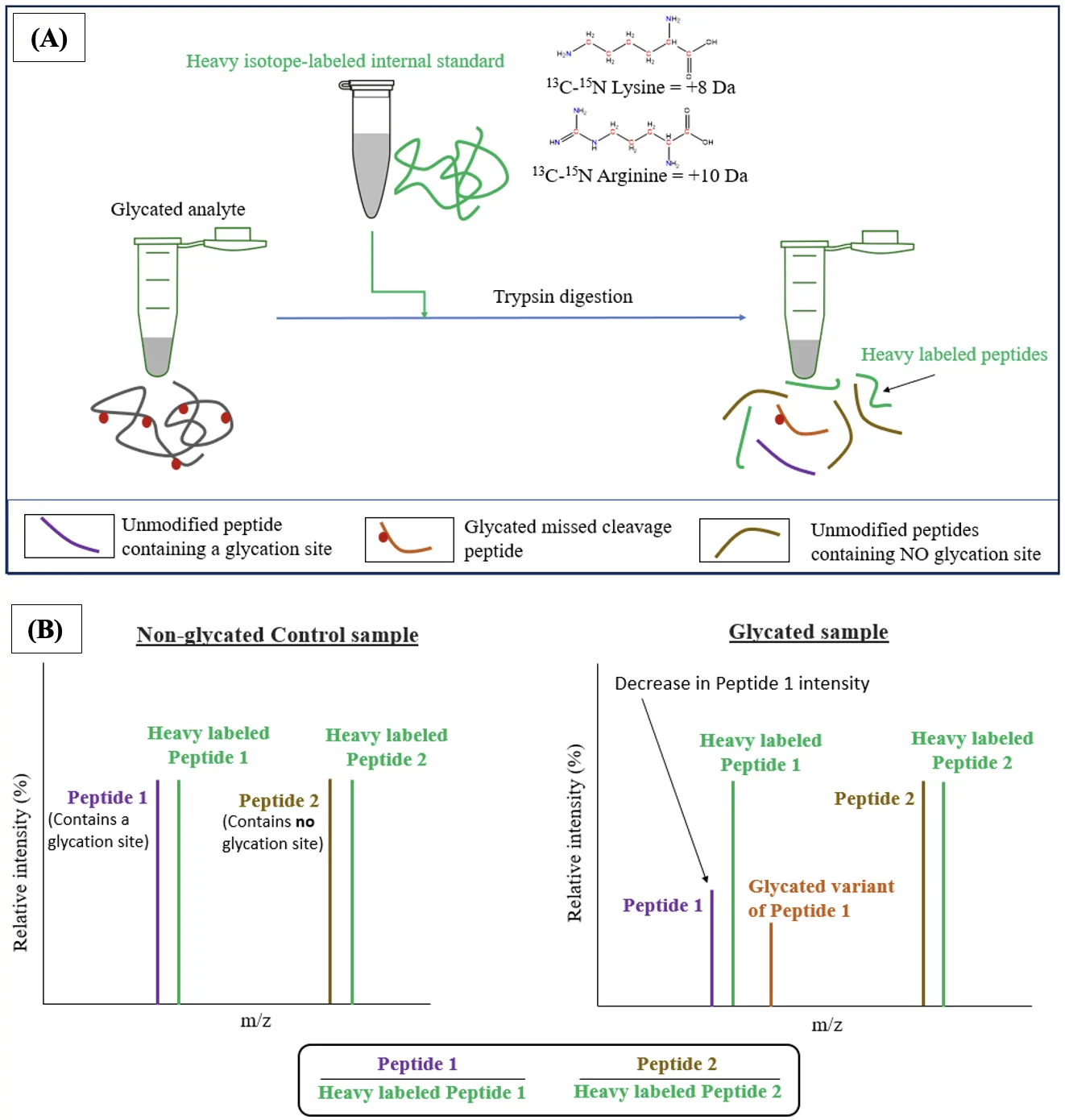
**Workflow for glycation quantitation using a ^13^C/^15^N lysine- and arginine-labeled protein internal standard.** (A) Illustration of glycation quantitation by using a heavy-isotope-labeled variant of the target protein. The heavy-isotope-labeled variant contained ^13^C and ^15^N lysine and arginine residues and was added before trypsin digestion to serve as an internal standard. The light/heavy ratio of unmodified tryptic peptides with a glycation site can be monitored to determine the extent of glycation at that site. (B) To develop the proposed method, IgGs were glycated in vitro to achieve detectable glycation. The light/heavy ratio of peptides with (Peptide 1) and without a glycation site (Peptide 2) was monitored to quantify glycation. A decrease in light/heavy ratio of Peptide 1 was observed owing to the formation of its glycated variant.

Herein, we propose using a nonglycated, heavy-isotope-labeled variant of the target protein to determine both the relative and absolute levels of glycation. A known amount of ^13^C and ^15^N lysine and arginine labeled protein could be added to the target protein before trypsin digestion (Figure 1). Quantitation can then be performed by monitoring the peak area of an unmodified tryptic peptide containing a glycation site relative to the peak area of its heavy isotope-labeled variant. This method offers several advantages. The characterization relies solely on the peak areas of tryptic peptides, rather than on those of potentially less abundant, glycated, or missed-cleavage peptides that exhibit poor ionization efficiency. The heavy-isotope-labeled variant of the target protein has physical and chemical properties comparable to those of the protein of interest, enabling accurate quantitation. The ionization efficiency of heavy-isotope-labeled peptides in mass spectrometry is similar to that of the corresponding light peptides in the protein of interest. Additionally, they exhibit similar retention times on an LC column, such as on hydrophilic interaction liquid chromatography (HILIC) column.^29^ Further, any variations in the sample processing steps, such as during enzymatic digestion, can be accounted for since the heavy-isotope-labeled internal standard is added before trypsin digestion, resulting in more reliable results.

To develop this method, IgGs such as adalimumab (AbbVie) (IgG1) and natalizumab (Biogen) (IgG4) were first forcibly glycated to achieve detectable glycation. Subsequently, their heavy-isotope-labeled variants containing ^13^C and ^15^N lysine and arginine residues were added, followed by trypsin digestion. A control IgG sample without glucose was processed similarly. HILIC chromatography, which has been previously employed to study hydrophilic modifications,^22,30–33^ was used in this study owing to the glycation product’s hydrophilic nature. It was hypothesized that the light/heavy ratio of a peptide containing a glycation site may decrease in the glycated sample compared to the nonglycated control due to the formation of its glycated variant (Peptide 1 in Figure 1B). Conversely, it was anticipated that the light/heavy ratio of a peptide lacking a glycation site (Peptide 2 in Figure 1B) would not change significantly when comparing the glycated sample to the nonglycated control. While demonstrated here for glycation, this framework is potentially applicable to other PTMs that alter peptide detectability or ionization efficiency.

## Materials and Methods

### In Vitro Glycation

IgGs such as adalimumab (AbbVie) (human IgG1) and natalizumab (Biogen) (IgG4 expressed in CHO cells) were obtained from GlycoScientific (Athens, GA, USA). In vitro glycation of these IgGs was performed by incubating the protein with a 1000 : 1 D-glucose : protein molar ratio in the presence of acetic acid (pH = 2.4), overnight at 65 °C. IgGs with no D-glucose were also subjected to the above-mentioned conditions to serve as a nonglycation control. D-glucose was removed from the reaction mixture by performing buffer exchange using Amicon Ultra-0.5 Centrifugal Filters (Merck Millipore Ltd., Burlington, MA, USA).

### Addition of Heavy-Isotope-Labeled Internal Standard

Heavy-isotope-labeled adalimumab (AbbVie) (IgG1) and natalizumab (Biogen) (IgG4) were obtained from GlycoScientific (Athens, GA, USA). These heavy-isotope-labeled variants were added to the glycated protein samples as well as to the nonglycation control samples in a 1:1 molar ratio. For instance, heavy-isotope-labeled IgG1 was added to the IgG1 glycated sample as well as to the IgG1 nonglycated control in a 1:1 molar ratio.

### Trypsin Digestion

After adding the heavy-isotope-labeled internal standard, the protein mixtures were buffer-exchanged into 50 mM ammonium bicarbonate (pH 7.8) to a final concentration of 1 mg/mL. They were then reduced using 200 mM dithiothreitol (DTT) and alkylated using 1 M iodoacetamide, both purchased from Sigma-Aldrich (St. Louis, MO, USA), to have a final concentration of 5 mM DTT and 8 mM iodoacetamide. Further, sequencing-grade trypsin purchased from Promega (Madison, WI, USA) was added at a 20:1 (w/w, protein/trypsin) ratio and incubated overnight at 37°C. The digested protein samples were then dried in a SpeedVac (Thermo Fisher Scientific, Waltham, MA, USA), resuspended in 80% ACN (1 mg/mL) and 20% H_2_O, and analyzed by LC–MS.

### LC-MS Settings and Instrumentation

Data were acquired on an Agilent 1100 series (Santa Clara, CA, USA) coupled to a Waters SYNAPT-G2 QTOF (Milford, MA, USA) system with an electrospray ionization (ESI) source operated in positive-ion mode. The ESI source was operated with a capillary voltage of 3.0 kV, a sampling cone voltage of 20 V, an extraction cone voltage of 3.0 V, and a source temperature of 120 °C. Peptides were separated using a 2.1-mm × 150-mm HALO Penta-HILIC column packed with 2.7-μm diameter superficially porous particles that have a 90-Å pore diameter (Advanced Materials Technology, Wilmington, DE, USA) at 60°C column temperature. The mobile phases used for separation were 50 mM ammonium formate in water with 0.1% formic acid (Solvent A) and 0.1% formic acid in acetonitrile (Solvent B). A linear gradient of 80% to 40% Solvent B over 40 minutes (1%B per minute) at 0.2 mL/min flow rate was used for separation. Data-dependent acquisition (DDA) survey-type experiments were run to characterize glycation. Mass spectral data analysis was carried out using Waters MassLynx (Milford, MA, USA), ProteinLynx Global Server (PLGS; Waters Corporation), and Skyline software (MacCoss Lab, University of Washington, Seattle, WA, USA). Extracted ion chromatograms (XICs) were generated using a mass width of ± 100 ppm.

## Results and Discussion

### Quantitation using Published Method

Glycation quantitation was first performed by calculating the ratio of the peak area of a glycated missed cleavage peptide to the total peak area of both the glycated peptide and the tryptic peptide it comprises, as described in the literature (Equation 1).^23^

(Eq. 1)% Relative glycation= Glycated peptide [Glycatedpeptide]+[Unmodified trypticpeptide constitutingglycated peptide]×100

Protein with substantial glycation was required to perform quantitation. Therefore, the study began with in vitro glycation of an IgG such as adalimumab (AbbVie) (human IgG1). The glycated sample was then enzymatically digested with trypsin, followed by LC-MS analysis on a Penta-HILIC column (Advanced Materials Technology, Wilmington, DE, USA) and a QTOF mass analyzer. Since glycation can inhibit the activity of trypsin,^20,22^ a missed tryptic cleavage, in conjunction with a 162 Da mass increase, was used as an indicator of glycation. The glycated missed cleavage peptides were confirmed using a HILIC retention coefficient developed for glycation PTM.^33^

**Figure 2.**
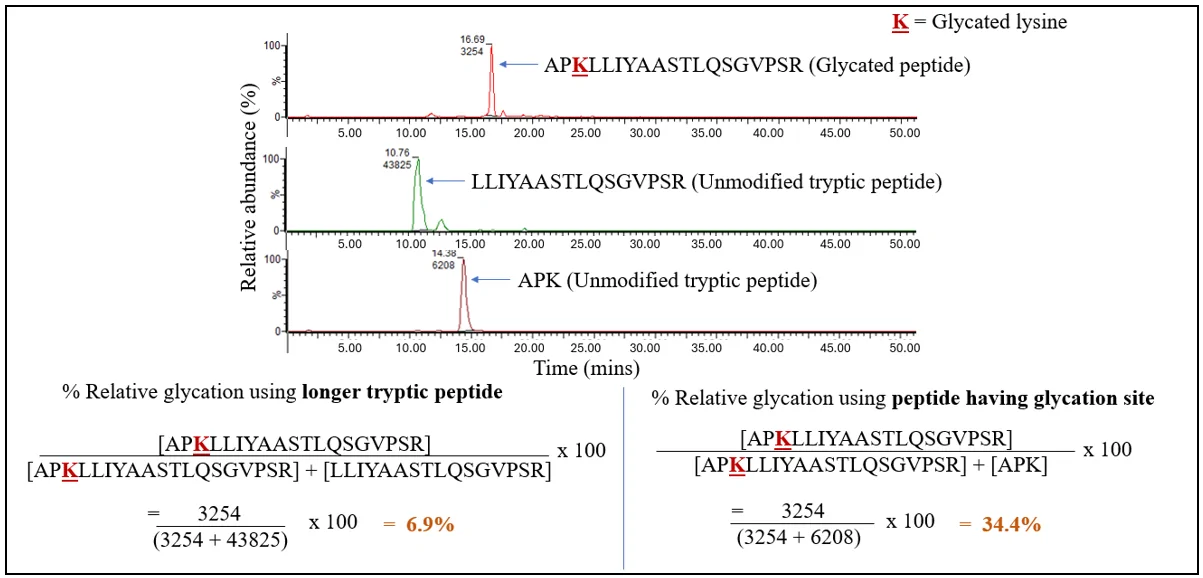
**Extracted ion chromatograms (XICs) of glycated peptide (APKLLIYAASTLQSGVPSR) and its unmodified counterparts (LLIYAASTLQSGVPSR and APK) in adalimumab (human IgG1).** Relative glycation (%) was performed using a published method. The percent glycation using this method was found to depend on the choice of unmodified peptide, yielding dramatically different results in both cases (6.9% versus 34.4%).

For instance, one of the glycated peptides observed in adalimumab (AbbVie) (human IgG1) was APKLLIYAASTLQSGVPSR. Quantitation could be performed at this site by using Equation 1, i.e., dividing the peak area of the glycated peptide APKLLIYAASTLQSGVPSR by the sum of the peak areas of the glycated peptide and its unmodified tryptic counterpart. The two tryptic peptides that constitute this glycated missed cleavage peptide are APK and LLIYAASTLQSGVPSR. The unmodified tryptic peptide to be used for calculation, as per the published method,^23^ could either be the longer of the two tryptic peptides that make up the glycated missed cleavage variant or the tryptic peptide that included the glycation site. LLIYAASTLQSGVPSR is the longer of the two peptides in this case, while APK is the tryptic peptide containing the glycation site. Thus, quantitation was performed using both methods, allowing inconsistencies to be examined (Figure 2). It was observed that the percent relative glycation was calculated to be 6.9% when the longer LLIYAASTLQSGVPSR tryptic peptide was used. On the other hand, the relative percent glycation was calculated to be 34.4% when using the APK tryptic peptide, which contained the glycation site. These results indicated that the accuracy of this method relied on the choice of the tryptic peptide used in the calculations. Another challenge associated with this approach was that the ionization efficiency of the glycated peptides was assumed to be comparable with that of the tryptic peptides. A larger glycated peptide with a missed cleavage, a glucose moiety attached, and a neutralized positive charge at the glycation site may exhibit lower ionization efficiency than a tryptic peptide. There may also be instances in which a glycated peptide coelutes with another tryptic peptide, making peak area integration challenging.

### Glycation Quantitation using Heavy-Isotope-Labeled Protein

It was hypothesized that a nonglycated, heavy-isotope-labeled variant of the target protein containing ^13^C- and ^15^N-labeled lysine and arginine residues could be used to quantify glycation. The heavy-labeled lysine was +8 Da compared to unlabeled lysine, and the heavy-labeled arginine was +10 Da compared to unlabeled arginine (Figure 1A). Heavy-isotope-labeled proteins with glycation modification are not commercially available; therefore, nonglycated heavy-isotope-labeled proteins could be utilized. It was anticipated that the abundance of unmodified peptides lacking a glycation site, i.e., those without a lysine residue and that are not N-terminus peptides, would be similar to that of their heavy-isotope-labeled variant. Alternatively, the light-to-heavy ratio of peptides containing a glycation site or adjacent to one may decrease after glycation. This decrease in the ratio could be extrapolated to determine the absolute and relative glycation at a specific protein site.

Glycation quantitation was performed on adalimumab (AbbVie) (human IgG1) to test this hypothesis. First, IgG1 was glycated to achieve observable glycation, then buffer-exchanged to remove excess glucose. Next, heavy-isotope-labeled IgG1 was added at a 1:1 molar ratio with unlabeled IgG1. This mixture was subjected to trypsin digestion. In parallel, a control IgG1 sample underwent in vitro glycation without glucose. An equal amount of heavy-isotope-labeled IgG1 (at a 1:1 ratio with control IgG1) was added prior to trypsin digestion. The control IgG1 sample was used to assess potential alterations in tryptic peptides in the absence of glucose.

To establish a reference for unmodified peptide behavior, light/heavy (L/H) ratios were calculated for peptides that met stringent selection criteria (absence of lysine residues or adjacency to lysine, non–N-terminal peptides, m/z >500, lack of co-eluting species, signal intensity >1000 counts, and lacking “NG” or “NN” motif that are prone to deamidation). The baseline L/H ratio was defined as the mean of these peptides across three replicates. Variability in the baseline was assessed by calculating the standard deviation and coefficient of variation (CV). Sensitivity of downstream glycation quantitation to baseline selection was evaluated by examining deviations in calculated glycation levels when the baseline ratio was perturbed by ±5–10%. No significant changes in relative glycation trends were observed within this range, supporting the robustness of the normalization approach.

**Figure 3.**
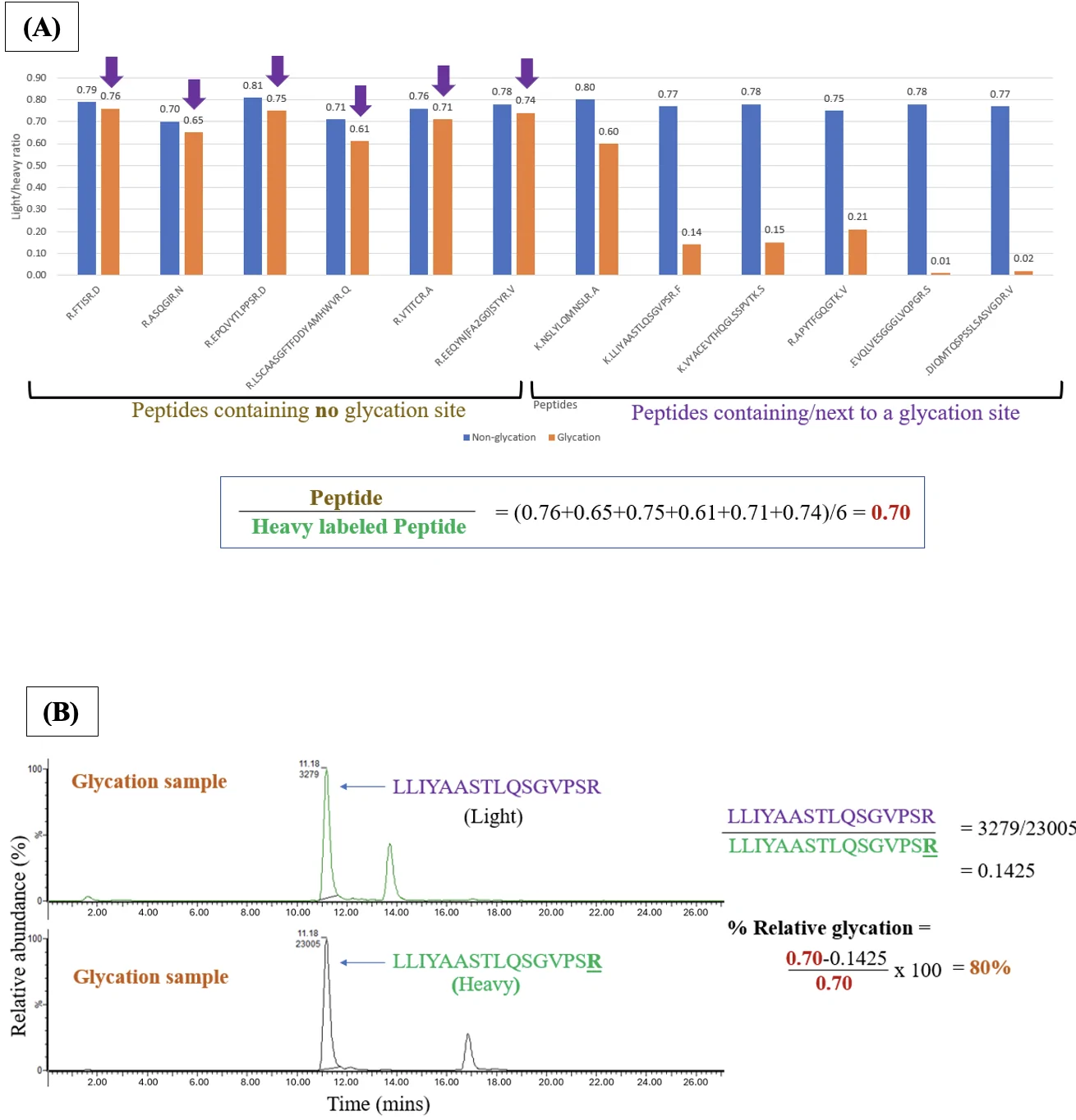
**Glycation quantitation on adalimumab using our proposed method.** (A) displays light/heavy ratios of peptides with and without a glycation site in adalimumab nonglycation control and glycation samples (from three replicates). The average light/heavy ratio for peptides that did not undergo significant modification was 0.70. (B) displays the XICs of unmodified light and heavy LLIYAASTLQSGVPSR tryptic peptides in the glycation sample run. % relative glycation at the APKLLIYAASTLQSGVPSR site was calculated to be 80% using the heavy-isotope-labeled internal standard approach. The APK tryptic peptide was not included in the calculation because its m/z is below 400 Da.

Tryptic peptides containing methionine were included because the light/heavy ratio of unmodified peptides in the glycation sample did not show a significant change compared with the nonglycation control. For example, the light/heavy ratios of FTISR, ASQGIR, EPQVYTLPPSR, LSCAASGFTFDDYAMHWVR, VTITCR, and EEQYN[A2G0F]STYR peptides in the IgG1 glycation sample were selected. The mean light/heavy ratio of these six peptides (Figure 3A) in the glycation sample was calculated to be 0.70 + 0.06, indicating consistent behavior among nonglycated peptides and justifying its use as a normalization baseline. Thus, if a peptide did not undergo glycation, its light/heavy ratio in the glycation sample is expected to be approximately 0.70. Any peptide with a decreased ratio would indicate a reduction in the unmodified peptide that could be attributed to glycation.

Peptides that contained a glycation site or were adjacent to one were analyzed. The light/heavy ratios of these peptides in the glycation sample ranged from 0.01 for EVQLVESGGGLVQPGR (N-terminus peptide) to 0.60 for NSLYLQMNSLR, indicating that glycation at these sites progressed to variable extents. Glycation quantification at the APKLLIYAASTLQSGVPSR site using our proposed heavy-isotope-labeled internal standard method is shown in Figure 3B. The light/heavy ratio for the LLIYAASTLQSGVPSR peptide in the glycation sample was calculated to be 0.1425, representing an 80% decrease from the previously calculated ratio of 0.70 (corresponding to the least modified peptides). This ~80% decrease in unmodified peptide abundance was interpreted as primarily reflecting glycation at this site under the experimental conditions. The APK tryptic peptide was not included in the calculation because its m/z is below 500 Da. Table 1A compares glycation quantification between the published method and our proposed method for an IgG1 sample across three replicates. It should be noted that the current study used a forced glycation model system for method validation and comparison between multiple approaches; these are not physiological values.

**Table 1.** Relative glycation (%) and corresponding % CV values for (A) adalimumab (human IgG1) and (B) natalizumab (IgG4) samples calculated using our proposed heavy isotope-labeled internal standard approach as well as the published method. Higher % relative glycation values were observed using the internal standard approach. This could be due to the poor ionization efficiency of the glycated peptides used in the published method. Alternatively, side reactions could consume unmodified tryptic peptide in the internal standard approach

**(A)**				
**Adalimumab (human IgG1)** **Peptides**	**Using Heavy-Isotope-Labeled Internal Standard Approach**		**Using Published Method**	
	**% Relative Glycation**	**CV (%)**	**% Relative Glycation**	**CV (%)**
VYACEVTHQGLSSPVTKSFNR	80%	2.5	37%	4.3
APKLLIYAASTLQSGVPSR	80%	4.1	APK- 34% LLIYAASTLQSGVPSR-7%	9.2 12.5
APYTFGQGTKVEIK	71%	2.9	35%	8.9
EVQLVESGGGLVQPGR	98%	2.4	41%	11.1
DIQMTQSPSSLSASVGDR	97%	2.2	39%	3.8
DNAKNSLYLQMNSLR	14%	7.1	Not detected	-
**(B)**				
**Natalizumab (IgG4)** **Peptides**	**Using Heavy-Isotope-Labeled Internal Standard Approach**		**Using Published Method**	
	**% Relative Glycation**	**CV (%)**	**% Relative Glycation**	**CV (%)**
EPQVYTLPPSQEEMTKNQVSLTCLVK	85%	2.8	14%	12.1
VYACEVTHQGLSSPVTKSFNR	43%	5.9	11%	10.0
EA**K**VQWK	72%	4.9	8.6%	8.7
TVAAPSVFIFPPSDEQL**K**SGTASVVCLLNNFYPR	82%	3.9	10%	6.0
DIQMTQSPSSLSASVGDR	87%	3.8	19%	5.6
LTVD**K**SR	94%	4.6	14%	10.7

It is worth noting that the tryptic peptide K.VYACEVTHQGLSSPVTK.S in IgG1 has a lysine residue both before it and at its C-terminus. Even so, the consumption of the VYACEVTHQGLSSPVTK peptide was attributed to glycation at the VYACEVTHQGLSSPVTKSFNR site since glycation at the lysine residue preceding the peptide was not detected. The percentage of relative glycation in IgG1 ranged from 14% at the DNAKNSLYLQMNSLR site to 98% at the EVQLVESGGGLVQPGR site, suggesting that this reaction was selective, with different sites undergoing glycation to varying extents. In addition, the relative glycation percentage obtained with our proposed heavy-isotope-labeled internal standard approach was higher than that calculated using the published method. This difference could be due to the under-representation of glycated peptides in the published method, as they may have lower ionization efficiency than tryptic peptides. Moreover, higher glycation values in our proposed approach may be due to other side reactions during glycation that could have consumed tryptic peptides.

**Figure 4.**
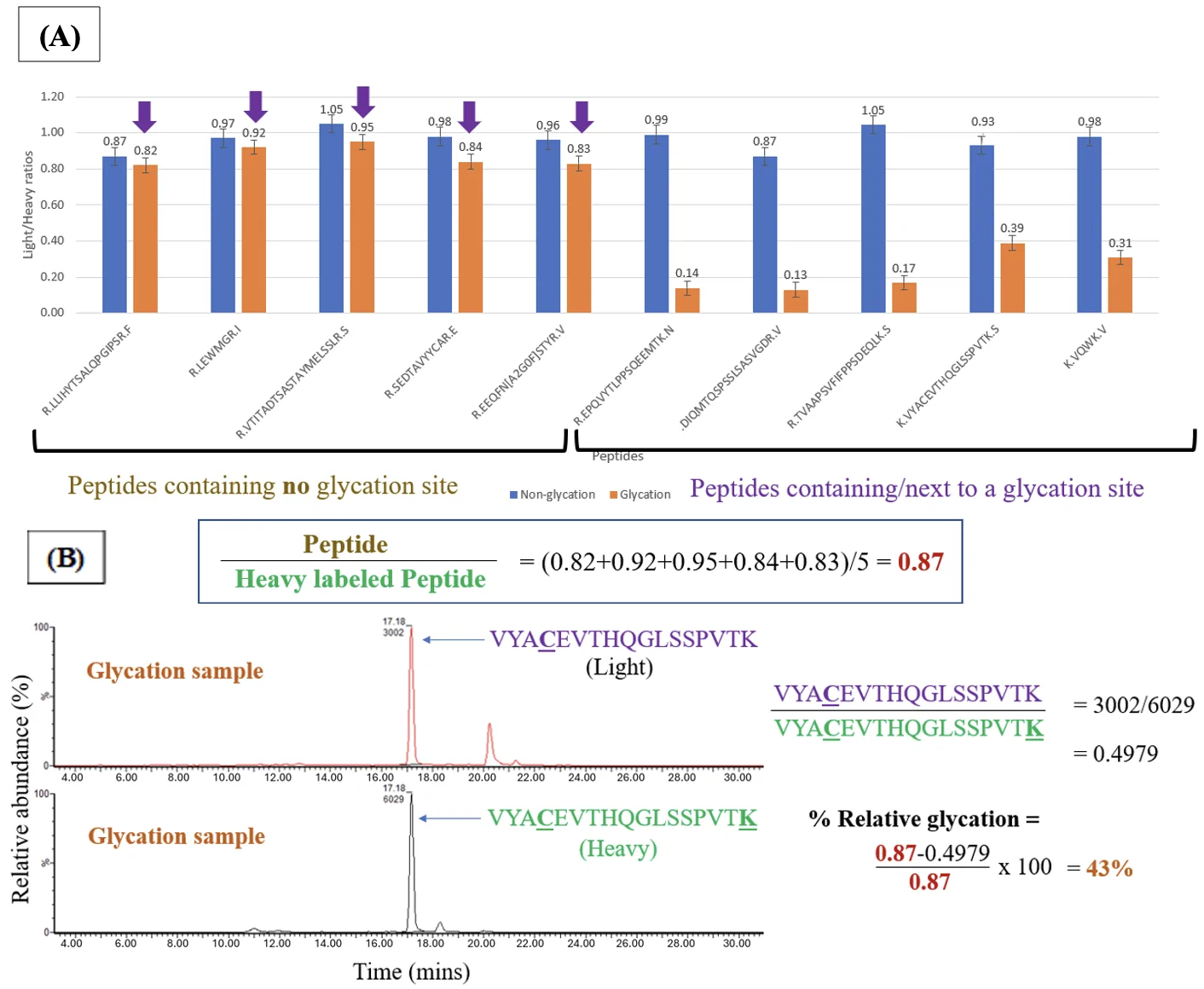
**Glycation quantitation on natalizumab (IgG4 expressed in CHO cells) using our proposed method.** (A) displays light/heavy ratios of peptides with and without a glycation site in natalizumab nonglycation control and glycation samples (from three replicates). The average light/heavy ratio for peptides that did not undergo significant modification was 0.87. (B) displays the XICs of unmodified light and heavy VYACEVTHQGLSSPVTK tryptic peptides in the glycation sample run. Relative glycation (%) at the VYACEVTHQGLSSPVTKSFNR site was calculated to be 43% using the heavy-isotope-labeled internal standard approach.

Glycation quantification of the natalizumab (Biogen) (IgG4) sample was performed next. The IgG4 sample was subjected to in vitro glycation as described previously along with a parallel control. Heavy-isotope-labeled IgG4 was added to both the nonglycation control and the glycation sample in a 1:1 molar ratio with the unlabeled protein. This mixture was subjected to trypsin digestion. Percentage of relative quantitation was performed using both the published method^23^ as well as the heavy-isotope-labeled internal standard approach. On average, the light/heavy ratio of peptides that did not contain or were adjacent to a glycation site from three replicates was calculated to be 0.87 (Figure 4A). The light/heavy ratio of peptides containing/next to a glycation site ranged from 0.13 for DIQMTQSPSSLSASVGDR (N-terminus peptide) to 0.39 for VYACEVTHQGLSSPVTK. As mentioned in the case of IgG1, the VYACEVTHQGLSSPVTK peptide has a lysine residue at the C-terminus and is adjacent to a lysine residue that precedes it. The consumption of this peptide was attributed to glycation at the VYACEVTHQGLSSPVTKSFNR site, as glycation at the lysine residue preceding this peptide was not detected. Similarly, consumption of the VQWK peptide was attributed to glycation at the EAKVQWK site since glycation at the lysine residue present at the C-terminus of this peptide was not detected. The light/heavy ratio for the VYACEVTHQGLSSPVTK peptide in the IgG4 glycated sample was 0.4979, suggesting a 43% glycation at this site (Figure 4B). Table 1B shows the % relative glycation determined using both the published method and the proposed heavy-isotope-labeled internal standard method.

To calculate the % relative glycation in a protein, a known amount of a heavy-isotope-labeled variant of the protein can be added, followed by trypsin digestion for a bottom-up approach. The light/heavy ratios of tryptic peptides that lack a glycation site and meet the previously mentioned criteria should be calculated and averaged. The light/heavy ratios of peptides containing or adjacent to a glycation site can then be calculated. Any decrease in these ratios relative to the averaged value for unmodified peptides can be attributed to relative glycation (%).

**Table 2.** Absolute glycation on (A) adalimumab (human IgG1) and (B) natalizumab (IgG4) samples. 6.76 μmol of heavy-isotope-labeled internal standard was used, which was normalized to 1 μmol. Absolute glycation was calculated by multiplying the light/heavy ratio of peptides by the normalized absolute amount of heavy-isotope-labeled internal standard used, followed by subtracting it from 1 μmol

**(A)**	
**Adalimumab (Human IgG1)**	**Absolute Glycation (μmol)**
VYACEVTHQGLSSPVTKSFNR	0.80
APKLLIYAASTLQSGVPSR	0.80
APYTFGQGTKVEIK	0.71
EVQLVESGGGLVQPGR	0.98
DIQMTQSPSSLSASVGDR	0.97
DNAKNSLYLQMNSLR	0.14
**(B)**	
**Natalizumab (IgG4)**	**Absolute Glycation (μmol)**
EPQVYTLPPSQEEMTKNQVSLTCLVK	0.85
VYACEVTHQGLSSPVTKSFNR	0.43
EA**K**VQWK	0.72
TVAAPSVFIFPPSDEQL**K**SGTASVVCLLNNFYPR	0.82
DIQMTQSPSSLSASVGDR	0.87
LTVD**K**SR	0.94

Absolute quantitation of the glycation PTM was performed by multiplying the light/heavy peptide ratio for peptides prone to glycation by the absolute amount of the heavy isotope-labeled internal standard (normalized to 1 µmol). This value, when subtracted from 1 µmol, yielded the absolute quantitation. Therefore, the absolute quantitation at the APKLLIYAASTLQSGVPSR site in IgG1 was determined by multiplying the light/heavy ratio of 0.1425 (Figure 3B) by 1 µmol, resulting in 0.1425 µmol. This value represented the amount of tryptic peptide remaining in the sample. The % absolute glycation at this site can then be calculated by subtracting 0.1425 from 1 µmol, resulting in 0.86 µmol. This calculation assumes equivalent digestion efficiency and recovery between light and heavy species and should therefore be interpreted as an approximate estimate of absolute glycation. Table 2 displays the absolute quantitation for (A) adalimumab (AbbVie) and (B) natalizumab (Biogen) from three replicates.

**Figure 5.**
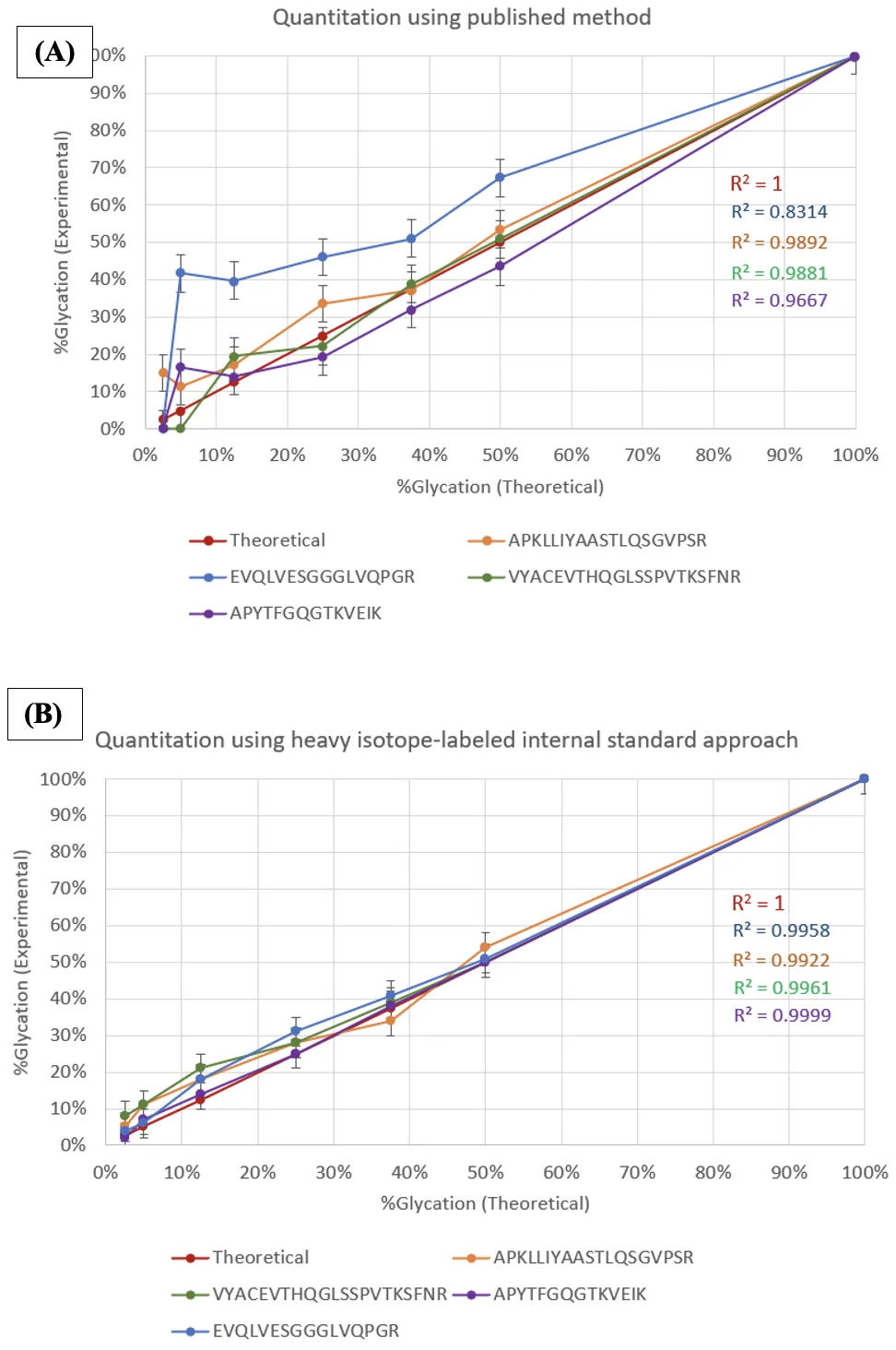
**Experimental vs. theoretical relative glycation (%) for dilution mixtures calculated using (A) the published method and (B) the proposed internal standard method.** A stronger correlation with the theoretical trend was observed with the heavy-isotope-labeled internal standard approach, indicating greater accuracy. Smaller error bars in the internal standard approach indicated improvement in precision.

Serial dilutions were performed to assess the linearity of our proposed heavy-isotope-labeled quantitation approach. Mixtures of nonglycated control and glycated adalimumab (AbbVie) (IgG1) were prepared at control:glycated ratios of 1:1, 1:0.75, 1:0.50, 1:0.25, 1:0.10, and 1:0.05, thereby decreasing the concentration of glycated species. These mixtures were then subjected to LC-MS analysis, and the detector’s response was recorded to verify conformity with the prepared dilutions. % Relative glycation for the undiluted glycated sample was normalized to 100%. Thus, % relative glycation at a particular site in the 1:1 mixture would theoretically be 50%. Glycation percentage would ideally be 37.5% in a 1:0.75 mixture, 25% in a 1:0.50 mixture, 12.5% in a 1:0.25 mixture, 5% in a 1:0.10 mixture, and 2.5% in a 1:0.05 mixture. Relative glycation (%) at different sites was calculated using both the published method and the heavy-isotope-labeled quantitation approach. The experimental relative glycation (%) in the undiluted glycated sample was normalized to 100%, and experimental results for the dilutions were adjusted accordingly. A plot of experimental vs. theoretical relative glycation (%) was created (Figure 5) for each method. The red trace on the plot depicts the ideal scenario: a linear trend with y = x. Figure 5A shows deviations from the ideal trend when the glycated peptides were quantified using the published method. Traces such as that of EVQLVESGGGLVQPGR deviated significantly from the ideal trend. Figure 5B depicts how the glycation quantitation correlated with the ideal trend when the heavy-isotope-labeled internal standard approach was used. The heavy-isotope-labeled approach showed improved agreement with the theoretical y = x relationship across all glycated peptides.

## Conclusions

This study presents a practical and reproducible strategy for the relative and absolute quantification of protein glycation using heavy-isotope-labeled full-length protein standards in a bottom-up LC-MS workflow. By incorporating the isotope-labeled version of the protein prior to digestion, the approach accounts for variability in sample handling and enzymatic processing while enabling quantitation based on the depletion of unmodified tryptic peptides rather than on the detection of low abundance glycated species.

Application of this method to monoclonal antibodies, including adalimumab (AbbVie) (IgG1) and natalizumab (Biogen) (IgG4), demonstrated consistent trends across multiple glycation sites and improved agreement with expected dilution behavior compared to a commonly used peak-area–based approach. These results highlight the advantages of using heavy-isotope-labeled protein standards to improve the robustness and precision of PTM quantitation.

The analytical performance of the proposed method was evaluated in terms of precision, linearity, and robustness. Across three replicates, the light/heavy ratios for peptides not susceptible to glycation remained consistent and exhibited low variability, indicating good reproducibility in sample processing and LC-MS analysis. The dilution series experiment further demonstrated strong agreement between experimental and theoretical glycation levels, supporting the method’s linearity over a broad range of glycation extents. Compared with a commonly used peak-area–based approach, the heavy-isotope-labeled internal standard method showed improved consistency with expected trends and reduced variability, suggesting enhanced quantitative reliability. While absolute accuracy could not be independently verified due to the lack of an orthogonal reference method, these results support the approach’s robustness and practical utility for glycation quantitation.

Importantly, glycation in this study was induced under controlled stress conditions to facilitate method development and evaluation. As such, the reported glycation levels reflect this experimental design and are intended to demonstrate method performance rather than represent typical levels observed in biotherapeutic products. Future work may extend this approach to more physiologically relevant or process-derived samples and further evaluate its analytical performance characteristics.

A key assumption underlying this approach is that reductions in the abundance of unmodified tryptic peptides are driven predominantly by glycation. While this is reasonable under controlled experimental conditions, other competing processes — such as side reactions during forced glycation, peptide degradation, or incomplete digestion — may also reduce peptide abundance. As a result, the method may overestimate glycation when these processes are significant. Future work incorporating orthogonal measurements, such as intact mass analysis or targeted detection of modified species, could further validate and refine this approach.

Overall, this study introduces a quantitative framework that shifts PTM analysis from direct measurement of modified species to indirect inference via depletion of unmodified peptides. By using full-length heavy-isotope-labeled protein standards, the method integrates correction for sample preparation variability and improves quantitative robustness. This strategy is broadly applicable and may be particularly valuable for PTMs that are analytically challenging due to low abundance or poor ionization efficiency.
